# Dentists’ readiness to accept an electronic oral health surveillance system in Egypt using a modified framework of the unified theory of acceptance and use of technology (UTAUT): a cross-sectional survey

**DOI:** 10.1186/s12903-024-05410-3

**Published:** 2025-01-16

**Authors:** Hams H. Abdelrahman, Maha Hamza, Wafaa Essam, May Adham

**Affiliations:** Department of Pediatric Dentistry and Dental Public Health, Faculty of Dentistry, Alexandria University, Champollion St, Azarita, 21526 Alexandria, Egypt

**Keywords:** Oral health surveillance, Mobile-oral health, E-health, Mobile technology, Health information technology, Acceptance model, UTAUT, Structural equation model

## Abstract

**Background:**

Effective public health surveillance is essential for policymaking and resource allocation. The World Health Organization (WHO) supports the integration of mobile technologies to create mobile Oral (m-Oral) Health surveillance systems to enhance disease monitoring. The effectiveness and sustainability of electronic health information initiatives depend on users’ acceptance of new technologies. This research assessed dentists’ acceptance of electronic oral health surveillance systems (EOHSS) and related factors, guided by the Unified Theory of Acceptance and Use of Technology (UTAUT) model.

**Materials and methods:**

A cross-sectional study included 1470 Egyptian dentists in an online survey from November 2023 to May 2024. The dentists were recruited from the five administrative regions in Egypt using convenience and snowball sampling. Participants responded to a questionnaire that was based on the UTAUT model. Structural equation model (SEM) was used for data analysis.

**Results:**

83.4% of dentists intended to use EOHSS. Performance expectancy (PE) (ß = 0.240, 95% CI: 0.182, 0.295), training adequacy (TA) (ß = 0.232, 95% CI: 0.165, 0.291), and effort expectancy (EE) (ß = 0.231, 95% CI: 0.169, 0.289) had the greatest influence on behavioral intention (BI). In contrast, anxiety towards electronic systems (ANX) (ß = -0.140, 95% CI: -0.187, -0.095) had a significant negative effect on BI. Effort Expectancy (EE) had a significantly stronger positive impact on BI of females than males. Moreover, EE had a significantly stronger impact on BI of dentists older than 40 years old than those who were younger than 30 years old.

**Conclusions:**

Egyptian dentists’ intentions to use the EOHSS were influenced by PE, TA, and EE. However, anxiety related to technology may limit its adoption. EE had a greater positive impact on BI in females and in older dentists.

**Supplementary Information:**

The online version contains supplementary material available at 10.1186/s12903-024-05410-3.

## Introduction

Timely data are crucial for guiding policy development, resource allocation, and interventions evaluation that improve health [1]. Disease surveillance systems use digital technology, significantly reshaping public health practice [2]. The World Health Organization (WHO) promotes the adoption of mobile technologies to establish mobile Oral Health (m-Oral Health) surveillance systems, facilitating coordinated and interoperable data gathering and monitoring [3]. These systems collect clinical data across geographically dispersed populations in an efficient, automated, and sustainable way [4], thus providing insights into the spatial distribution and temporal progress of diseases [5]. Electronic surveillance systems provide up-to-date data at low cost while mitigating the risks of data duplication and manipulation, offering immediate analysis and access to policymakers [6]. 

The adoption of digital health information systems is scarce in low- and middle-income countries. Only 35% of lower-middle-income (LMICs) and 15% of low-income countries have successfully implemented these systems [7]. This may be attributed to personal, technological, social, and organizational factors including administrative challenges, and resistance to embracing new technologies [8]. The implementation and sustainability of electronic health information projects hinge on the acceptability of these new technologies by organizations and end users [9]. Therefore, identifying this acceptability levels of users before deployment is essential for planning and designing effective implementation strategies and preventing resource waste [10]. Information technology adoption theories aids in predicting and understanding how end-users will respond, supporting successful implementation [11]. 

Various theoretical models were introduced to explain technology adoption. Among these, the Unified Theory of Acceptance and Use of Technology (UTAUT) is a well-recognized model with higher explanatory power than other technology acceptance theories [12]. The UTAUT model suggests that behavioral intentions (BI) significantly influence user’s acceptance and use of technology. Four key constructs positively affect the BI: performance expectancy (PE), effort expectancy (EE), social influence (SI), and facilitating conditions (FC) [13, 14]. PE is the degree to which a person believes that adopting the new technology will improve job performance, while EE is the perceived ease of using the new technology. Social influence involves the influence of reference individuals on the use of technology, and FC concerns the availability of organizational or infrastructural resources that support its use [15]. 

In low resource settings, the classical UATUT model may not fully explain healthcare technology adoption [15, 16]. Healthcare professionals (HCPs) in these settings have negative attitude toward using innovative technology, low digital literacy, and experience, therefore, context-specific variables that need to be incorporated in the model to strengthen its explanatory power. These may include training adequacy (TA) [17], technology anxiety (ANX) [18–20], and resistance to change (RC) [18, 21]. 

Training adequacy is the degree to which users perceive their training to be enough to use new technology effectively while resistance to change describes individuals’ reluctance to alter their current state, due to organizational or personal factors. Technology anxiety describes the anxiety, fear, and unease that some individuals experience when interacting with technology [22]. Training adequacy is assumed to positively impact the acceptance of technology, while resistance to change and technology anxiety are barriers to the successful transition to digital health information systems [17–20]. Since the system has not yet been implemented in Egypt, it is important to address challenges related to users’ anxiety and prejudices about potential declines in their work performance when new technologies are introduced. Additionally, assessing training adequacy is crucial for identifying training gaps that may hinder adoption, allowing for focused efforts to support a smoother implementation process [17, 18, 23]. 

Egypt has embarked on an agenda of digital transformation in the health sector led by the Ministry of Health and Population (MOHP) in alignment with Egypt’s 2030 vision [24]. The study aims to evaluate the acceptability of an electronic oral health surveillance system (EOHSS) by Egyptian dentists, and to assess the factors influencing their intentions to use the system. Based on the modified UTAUT model, it was hypothesized that performance expectancy, effort efficiency, social influence, facilitating conditions, and training adequacy would have positive influence on behavior intention to adopt the EOHSS while resistance to change and technology anxiety would negatively impact EOHSS adoption. This study will provide system developers and implementers of EOHSS with insights to address potential issues, facilitating the successful design and deployment of EOHSS in Egypt and other developing countries.

## Methods

### Study design and settings

The study was conducted using a cross-sectional survey from November 2023 and May 2024. The Faculty of Dentistry’s Research Ethics Committee at Alexandria University in Egypt granted ethical approval (#0724-7/2023). The Declaration of Helsinki’s guiding principles were followed, and the STROBE guidelines were used to report the current study.

### Study participants and eligibility criteria

The study included all licensed Egyptian dentists, employed in the public sector, who are expected to be EOHSS users. This was explicitly stated at the beginning of the survey, highlighting that our target demographic only consists of dentists employed within the governmental sector excluding students, interns, retired dentists, and dentists worked only in the private practice.

### Sample size and sampling procedures

The sample size was calculated in accordance with the established guideline recommending a minimum of 10 to 20 participants per parameter in structural equation modeling [25]. The proposed model involved 50 parameters including 35 factor loadings, eight latent variables and seven coefficients of exogenous and endogenous variables. Consequently, the required sample size was 1,000. To account for 50% non-response rate, the final sample size was 1,500 dentists. The sample was proportionally allocated to the five geographic regions of Egypt: Greater Cairo, Alexandria area, Delta area, Suez Canal area, and Upper Egypt [26]. Convenience and snowball sampling were used for participants recruitment. [Additional File 1]

### Questionnaire design and pilot testing

**Fig. 1:**
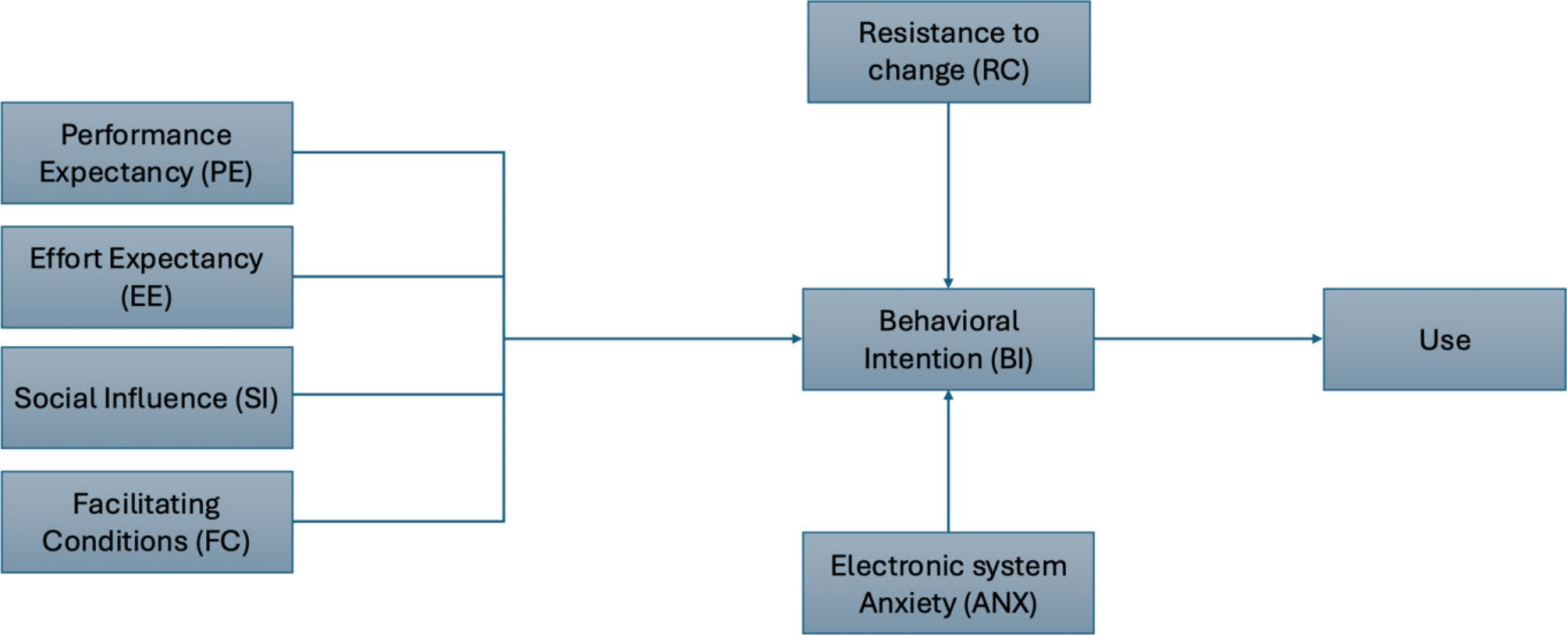
Conceptual framework for EOHSS acceptance

A modified UTAUT model was used to design a self-administered questionnaire [18, 27–29]. [Figure 1] The questionnaire had two sections; Section 1 gathered demographic information, including age, gender, the governorate of workplace, educational level, years of professional experience, sector of employment (public (governmental) or private), type of workplace, and geographical classification (urban or rural). Section 2 encompassed 35 questions addressing eight constructs including six PE questions, four EE questions, three SI questions, five FC questions, four TA questions, seven ANX questions, two RC questions and three questions assessing the intention to use the EOHSS. A five-point Likert scale, with responses ranging from (1) “strongly disagree” to (5) “strongly agree,” was used to assess agreement with the items. Items measuring resistance to change were reverse-coded. [Additional file 2]

Before the study, content validity of the items was evaluated by nine dental academics to judge their relevance on a four-point scale ranging from (1) not relevant to (4) highly relevant. The Item-level Content Validity Index (CVI-I) was calculated by dividing the number of experts who rated the items with scores of three or four by number of experts. The CVI-I score was 0.91, indicating acceptable validity [30]. Twelve MOH dentists assessed the clarity and comprehensibility of items. Dentists who participated in the pre-testing were not included in the study sample.

### Data collection

An anonymous questionnaire was distributed via the online survey platform “Survey Monkey” to Egyptian dentists’ professional groups on social media, including LinkedIn, Facebook, Instagram, and WhatsApp. These groups were on the official pages of dental syndicates, scientific associations and web pages of postgraduate students at various Egyptian universities. Also, dentists in various governorates were asked to disseminate the survey in their networks and to post it in official closed WhatsApp groups used to share updates and work-related information. Respondents were permitted to modify their answers before submission, and duplicate entries were prohibited. Completing the questionnaire typically required between 9 and 13 min. At the beginning of the questionnaire, the study purpose was explained, participants were informed that their participation was voluntary, that they had the right to withdraw from the study at any time and that no personal identifiers were collected. It was also clarified that no responses were considered right or wrong, to foster unbiased participation. No monetary remunerations were offered,

### Statistical analysis

SPSS version 24.0 (IBM Corp., Armonk, NY, USA) was used for analysis. The intention to use the EOHSS was assessed by calculating the sum of scores of the three BI questions. Participants who scored at or above the median were considered to have the intention to use the EOHSS [31, 32]. To determine the suitability of the data for Principal Component Analysis (PCA), the following tests were calculated: the Kaiser-Meyer-Olkin (KMO) measure of sampling adequacy and it was compared with the recommended threshold of 0.6 and the Bartlett’s test of Sphericity was checked for whether it was statistically significant [33]. Validation of the UTAUT model was based on a path analysis with structural equation modeling using a partial least squares model (SEM-PLS) in SmartPLS 4.1.0.6 (SmartPLS GmbH, Boenningstedt, Germany). Internal reliability was assessed using Cronbach’s alpha (α) where α >= 0.70 indicates acceptable consistency [34]. Convergent validity was evaluated by the Average Variance Extracted (AVE), where items loadings with AVE > = 0.50 demonstrate construct validity. Discriminant validity was evaluated using the Fornell-Larcker Criterion. This involves ensuring that the square root of the AVE is greater than the correlations with other constructs and that diagonal elements of the matrix exceed the off-diagonal elements. The Variance Inflation Factor (VIF) was calculated to assess multicollinearity. VIF < 5 indicate minimal concerns about multicollinearity [34]. To assess the overall goodness of fitness, Standardized Root Mean Square (SRMR) and Normal Fit Index (NFI) were employed. An acceptable model fit is indicated by SRMR < 0.05 and NFI > 0.90. R^2^ was calculated to assess how well the variables in the model explain the variation in the dependent variable (BI). Stone-Geisser Q^2^ value was calculated to assess the predictive relevance of the model that should be above 0 [35, 36]. Bootstrapping with 5,000 subsamples was used to test the significance of the relationships between latent variables, using two-tailed t-statistic with a significance threshold of 0.05. The estimates of the path coefficients were calculated to show the direction, magnitude, and significance of the relationships in the structural model. Venkatesh et al. [37], proved that age and gender affect BI. For moderation analysis, multi-group analysis was used where gender was stratified by male and female and age was stratified into under 30 years, 30 to 40 years, and over 40 years. Sociodemographic factors beyond age and gender were excluded as moderators because their inclusion reduced model fit.

## Results

### Characteristics of participants

A total of 1512 dentists were surveyed, with 1470 valid responses (response rate = 97.2%). Most respondents were under 40 years of age (81.5%), 60.4% were female, 58.0% had 10 years or less of work experience and 59.0% held a Bachelor of Dental Surgery (BDS) degree. Also, 59.6% worked in primary healthcare units and 79.0% worked in urban areas. [Table 1]

**Table 1:** Demographic characteristics of the Egyptian dentists working in governmental sectors

Variables		Total sample = 1470
Age Categories (years)	20–29	558 (38.0%)
	30–39	640 (43.5%)
	40–49	138 (9.4%)
	50–60	134 (9.1%)
Gender	Males	582 (39.6%)
	Females	888 (60.4%)
Qualifications	BDS	868 (59.0%)
	Diploma	230 (15.6%)
	MSc	320 (21.8%)
	PhD	52 (3.5%)
Work Experience	< 5 years	442 (30.1%)
	5–10 years	410 (27.9%)
	> 10–20 years	424 (28.8%)
	> 20 years	194 (13.2%)
Sector	Primary Health Care Units	876 (59.6%)
	General Hospitals	398 (27.1%)
	Educational Hospitals	196 (13.3%)
Geographical Location	Urban	1162 (79.0%)
	Rural	308 (21.0%)

### Intention to use EOHSS

The majority (83.4%, *n* = 1226) expressed an intention to use EOHSS, with median BI score = 12 out of 15, with a range from 3 to 15.

### Model validity and fit

Following the initial evaluation of model validity and fit, the items crossed out in Additional File 2 were excluded because their alpha values were < 0.7 and AVE > 5, indicating inadequate reliability and validity. Following this, SmartPLS was rerun with the remaining items, resulting in a final model of seven latent variables: PE, EE, SI, FC, TA, RC, ANX, and 24 items in addition to BI. In the final model, the KMO = 0.899, and the *p* value of Bartlett’s test < 0.001. SRMR = 0.047, NFI = 0.908 and R^2^ = 50.6% indicating good model fit. Additionally, Q^2^ value = 0.501 indicating robust ability to explain variability in the constructs examined. All constructs exhibited item loadings > 0.7, AVE values > 0.5 and alpha values > 0.7, and indicating satisfactory reliability and convergent validity. The VIF ranged between 1.536 and 4.188, indicating no multicollinearity problems. These strong psychometric results offer a reliable basis for subsequent structural equation modeling. [Table 2] The diagonal of Table 3 shows that the square root values of AVE were > the correlations among the inner constructs in columns and rows, indicating good discriminant validity.

**Table 2:** Model reliability and measures of Convergent Validity

Construct	Items	Loadings	VIF	AVE	CR	Cronbach’s alpha
Performance Expectancy (PE)	PE1	0.856	2.305	0.684	0.849	0.845
	PE2	0.878	2.561			
	PE3	0.808	1.833			
	PE4	0.763	1.536			
Effort Expectancy (EE)	EE1	0.901	2.714	0.817	0.890	0.888
	EE2	0.918	2.887			
	EE3	0.892	2.288			
Social Influence (SI)	SI1	0.861	1.684	0.752	0.851	0.837
	SI2	0.886	2.508			
	SI3	0.854	2.180			
Facilitating Conditions (FC)	FC1	0.777	1.677	0.686	0.867	0.848
	FC2	0.832	2.009			
	FC3	0.840	2.161			
	FC4	0.861	2.053			
Training Adequacy (TA)	TA1	0.895	2.643	0.810	0.883	0882
	TA2	0.927	3.197			
	TA3	0.877	2.151			
Resistance to change (RC)	RC1	0.905	1.686	0.819	0.779	0.779
	RC2	0.905	1.686			
Anxiety toward electronic systems (ANX)	ANX1	0.907	2.321	0.790	0.879	0.867
	ANX2	0.889	2.304			
	ANX3	0.869	2.172			
Behavioral Intention (BI)	BI1	0.941	3.921	0.886	0.936	0.936
	BI2	0.943	4.188			
	BI3	0.940	3.995			

KMO = 0.891, *P* value of Bartlett’s test < 0.001, VIF: Variance Inflation Factor, AVE: Average Variance Extracted, CR: Composite Reliability

**Table 3:** Discriminant Validity between constructs using Fornell – Larcker Criterion

	PE	EE	SI	FC	TA	RC	ANX	BI
**PE**	**0.827**							
**EE**	0.520	**0.904**						
**SI**	0.393	0.363	**0.867**					
**FC**	0.362	0.405	0.251	**0.828**				
**TA**	0.452	0.437	0.299	0.680	**0.900**			
**RC**	-0.121	-0.086	0.068	-0.048	-0.088	**0.905**		
**ANX**	-0.240	-0.422	-0.118	-0.178	-0.186	0.257	**0.889**	
**BI**	0.559	0.576	0.358	0.449	0.539	-0.145	-0.370	**0.941**

PE: Performance Expectancy, EE: Effort Expectancy, SI: Social Influence, FC: Facilitating Conditions, TA: Training Adequacy, RC: Resistance to Change, ANX: Technology Anxiety, BI: Behavior Intention

### Structural equation model

**Fig. 2:**
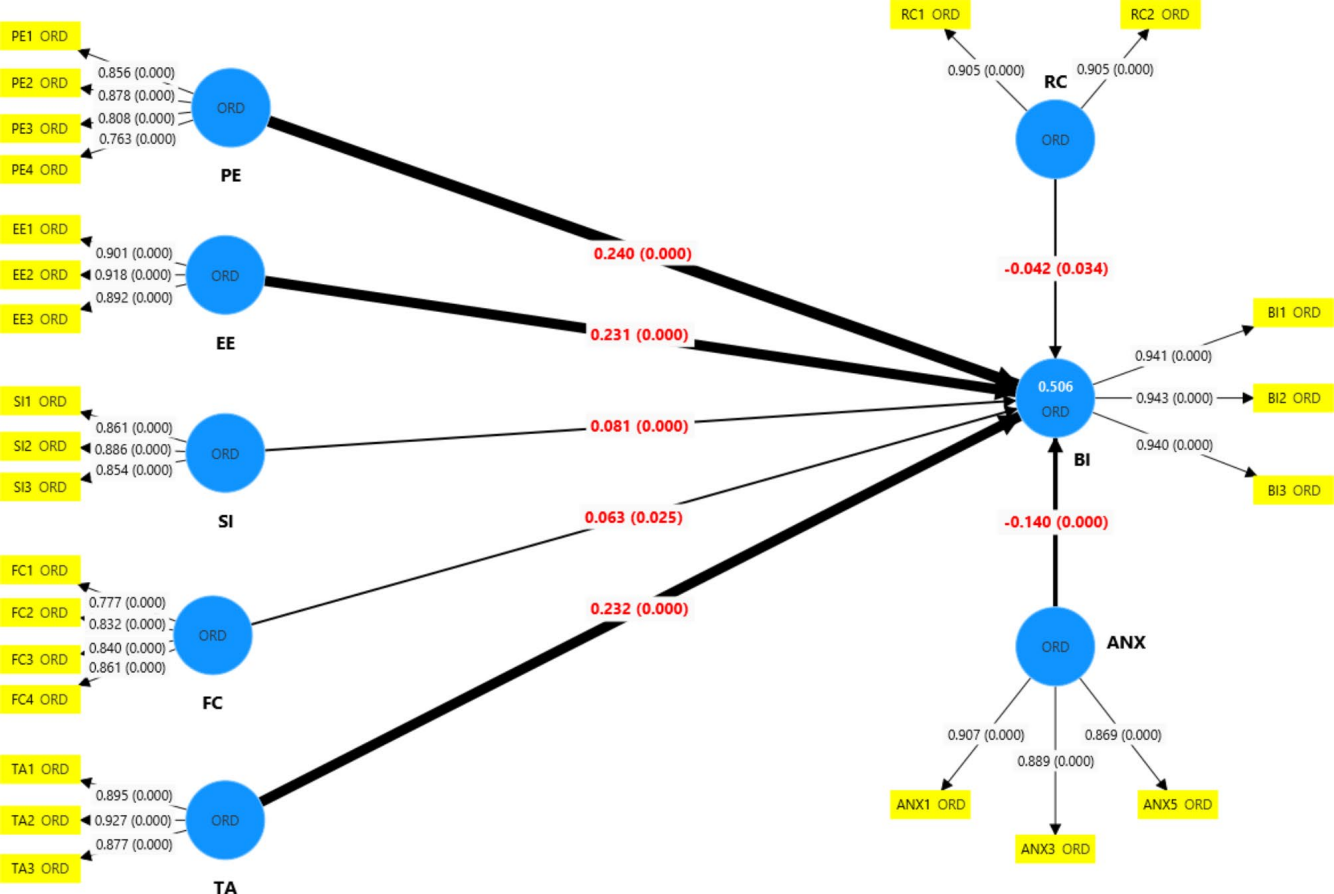
UTAUT modified model of constructs affecting intention to use EOHSS among dentists in Egypt. Yellow boxes represent observed variables and blue circles denote latent variables. The values in red are path coefficient estimates, with *p*-values shown in brackets. Thick path lines denote large coefficient estimates. PE: Performance Expectancy, EE: Effort Expectancy, SI: Social Influence, FC: Facilitating Conditions, TA: Training Adequacy, RC: Resistance to Change, ANX: Technology Anxiety, BI: Behavior Intention

Table 4; Fig. 2 show that performance expectancy had the greatest positive effect on BI (ß = 0.240, 95% CI: 0.182, 0.295, *p* < 0.001), followed by training adequacy (ß = 0.232, 95% CI: 0.165, 0.291, *p* < 0.001), and effort expectancy (ß = 0.231, 95% CI: 0.169, 0.289, *p* < 0.001). Social influence (ß = 0.081, 95% CI: 0.039, 0.127, *p* < 0.001) and facilitating conditions (ß = 0.063, 95% CI: 0.011, 0.122, *p* < 0.001) had significant weak positive effect on BI. Technology anxiety and resistance to change had significant negative effect on BI (ß = -0.140, 95% CI: -0.187, -0.095, *p* < 0.001 and ß = -0.042, 95% CI: -0.083, -0.005, *p* < 0.001). The latent variables explained 50.6% (95% CI: 46.3, 55.4) of the variance in BI.

**Table 4:** Multiple structural equations modeling association between predictors and intention to use EOHSS among Egyptian dentists

Path	ß	95% CI	t-statistic	*p* value
PE → BI	0.240	0.182, 0.295	8.403	< 0.001*
EE → BI	0.231	0.169, 0.289	7.568	< 0.001*
SI → BI	0.081	0.039, 0.127	3.589	< 0.001*
FC → BI	0.063	0.011, 0.122	2.239	0.025*
TA → BI	0.232	0.165, 0.291	7.207	< 0.001*
RC → BI	-0.042	-0.083, -0.005	2.123	0.034*
ANX → BI	-0.140	-0.187, -0.095	5.996	< 0.001*

*Statistically significant difference at *p* value < 0.05, ß: Beta Coefficient, CI: confidence interval, PE: Performance Expectancy, EE: Effort Expectancy, SI: Social Influence, FC: Facilitating Conditions, TA: Training Adequacy, RC: Resistance to Change, ANX: Technology Anxiety, BI: Behavior Intention

### Moderation effect of age and gender

*E*xcept for effort expectancy, the impact of all latent variables on BI did not significantly differ by gender. The effect of effort expectancy in male dentists, ß= 0.148, *p* = 0.002, was significantly less in females, ß= 0.280, *p* < 0.001, *p* value for the difference between genders was 0.038. [Table 5] The influence of all latent variables on BI did not differ significantly between dentists in the two younger age groups. By contrast, there were significant differences between dentists under 30 years and those over 40 years in the effect of performance and effort expectancy, facilitating conditions, resistance change and technology anxiety on BI. However, only the effect of effort expectancy on BI was significant in dentists < 30-year-old (ß= 0.270, *p* < 0.0001) and > 40-year-old (ß= 0.441, *p* < 0.0001) with a significant difference between both (*p* = 0.027). [Table 6]

**Table 5:** Moderating effect of gender on dentists’ BI to adopt EOHSS

Path	Gender	ß	t-statistics	*P* value	Difference	*p* value
PE → BI	Male	0.276	5.529	< 0.001*	0.049	0.409
	Female	0.227	6.902	< 0.001*		
EE → BI	Male	0.148	3.035	0.002*	-0.132	0.038*
	Female	0.280	6.932	< 0.001*		
SI → BI	Male	0.066	1.909	0.056	-0.031	0.493
	Female	0.097	3.202	0.001*		
FC → BI	Male	0.084	1.883	0.060	0.040	0.482
	Female	0.044	1.267	0.205		
TA → BI	Male	0.287	5.436	< 0.001*	0.093	0.160
	Female	0.193	4.804	< 0.001*		
RC → BI	Male	-0.017	0.551	0.582	0.049	0.212
	Female	-0.066	2.562	0.010*		
ANX → BI	Male	-0.123	3.636	< 0.001*	0.018	0.701

*Statistically significant difference at *p* value < 0.05, ß: Beta Coefficient, Males model: R^2^ of BI = 0.535, Females model: R^2^ for BI = 0.486, PE: Performance expectancy, EE: Effort expectancy, SI: Social Influence, FC: Facilitating Conditions, TA: Training Adequacy, RC: Resistance to change, ANX: Technology anxiety

**Table 6:** Moderating effect of age on dentists’ BI to adopt the EOHSS

Path	Age Group	ß	t-statistics	*P* value	Difference	*p* value
PE → BI	< 30	0.269	6.013	< 0.001*	Ref	
	30–40	0.301	7.071	< 0.001*	0.031	0.611
	> 40	0.033	0.660	0.509	-0.238	< 0.001*
EE → BI	< 30	0.270	6.103	< 0.001*	Ref	
	30–40	0.145	3.05	0.002*	-0.124	0.056
	> 40	0.441	6.884	< 0.001*	0.173	0.027*
SI → BI	< 30	0.114	3.227	0.001*	Ref	
	30–40	0.054	1.640	0.101	-0.060	0.212
	> 40	0.071	1.374	0.170	-0.043	0.486
FC → BI	< 30	0.028	0.657	0.511	Ref	
	30–40	0.045	1.032	0.302	0.017	0.782
	> 40	0.213	2.984	0.003*	0.185	0.026*
TA → BI	< 30	0.184	3.639	< 0.001*	Ref	
	30–40	0.295	6.087	< 0.001*	0.111	0.113
	> 40	0.078	1.098	0.272	-0.106	0.224
RC → BI	< 30	-0.032	1.067	0.286	Ref	
	30–40	-0.005	0.158	0.875	0.027	0.532
	> 40	-0.230	3.962	< 0.001*	-0.198	0.003*
ANX → BI	< 30	-0.140	3.597	< 0.001*	Ref	
	30–40	-0.169	5.226	< 0.001*	-0.03	0.557
	> 40	0.038	0.761	0.447	0.178	0.005*

*Statistically significant difference at *p* value < 0.05, ß: Beta Coefficient, , > 30 years model: R^2^ of BI = 0.531, 30–40 years model: R^2^ for BI = 0.520, > 40 years model: R^2^ for BI = 0.529, PE: Performance expectancy, EE: Effort expectancy, SI: Social Influence, FC: Facilitating Conditions, TA: Training Adequacy, RC: Resistance to change, ANX: Technology anxiety

## Discussion

The study showed that most dentists intended to use the EOHSS, and this intention was associated with the UTAUT model constructs which, thus, explained half the variance in behavior intention. Performance expectancy, effort expectancy and training adequacy had a strong positive impact on behavior intention and technology adequacy had the strongest negative impact. The effect of effort expectancy was moderated by gender and differed significantly between < 30 and > 40-year-old dentists.

Performance expectancy had the strongest positive effect on behavioral intention, which is consistent with studies from Ethiopia [8], Cameroon [15, 28], and Ghana [38]. In these countries, healthcare workers’ intention to adopt health information systems was significantly influenced by the perceived usefulness of these systems, particularly their ability to improve work efficiency, and support data-driven decisions. These findings highlight the benefits of such systems in LMICs [8, 15, 38, 39]. Also, Egyptian dentists intend to use the EOHSS to enhance their work performance, by recording data more efficiently, reducing workload and errors and addressing time-consuming manual data management [40, 41]. Moreover, new electronic systems may stimulate motivation and provide learning opportunities, potentially enhancing dentists’ prospects for promotion. Egypt’s Vision 2030 aims to modernize the healthcare sector, the alignment of performance expectancy with career development is particularly relevant. The healthcare system increasingly seeks professionals proficient in digitalization to propel the nation’s digital transformation [42]. By emphasizing performance-based incentives, healthcare organizations can motivate workers to adopt EOHSS [43]. 

Effort expectancy positively affected BI which aligns with previous research from Ethiopia [2, 8, 40], Tanzania [17], and Ghana [38] assessing the acceptance of electronic health records and mobile health information applications. This could be attributed to users’ inclination toward systems that are user friendly and practical, to improve workflow efficiency and data management, which enhances overall productivity and ease of use. Thus, organizations should prioritize positive user experience when developing EOHSS so that dentists find it intuitive and easy, and better perceive its benefits, leading to greater acceptance.

Training adequacy and facilitating conditions were significant predictors of EOHSS acceptance, with training adequacy emerging as strong positive predictor. The dentists acknowledged the importance of having electronic devices and technical support available for EOHSS in their workplace. However, they also perceived adequate training as a critical enabler for the efficient use of the new system which mirrors findings from several developing countries [8, 27, 39]. In many LMICs, barriers such as poor internet connectivity, underdeveloped digital infrastructure, and limited funding for training programs hinder the adoption of systems like EOHSS. Addressing these deficiencies is essential to creating an enabling environment that supports the implementation and utilization of such technologies [8, 27, 39]. 

Facilitating conditions is strongly supported by Egypt’s Vision 2030, which prioritizes healthcare modernization through investments in digital infrastructure, including expanded fiber optic networks, internet accessibility, telemedicine, AI, and cloud-based systems in health facilities [44]. These initiatives create an environment conducive to the EOHSS deployment and acceptance. Despite the push for digital transformation, a comprehensive training plan to prepare dentists for this shift is still to be implemented. Current training of HCPs focuses on clinical skills, with minimal emphasis on digital literacy and competency in using digital health tools effectively which limits the adoption of new technologies [45]. Several training initiatives are currently in their early stages. For example, the Health Information Technology Center, of the MOHP, offers online courses in health information technology and health informatics. These courses are freely accessible, self-paced, but not mandatory for healthcare providers [46]. Moreover, the MOHP in collaboration with the WHO, has launched preliminary actions focused on health data analytics and big data techniques [47, 48]. However, these training programs are not widely accessible to healthcare professionals, limiting their potential impact.

Implementing online and in-person, targeted training programs for HCPs across Egypt would help develop knowledge and skills in using the EOHSS. Given the high costs of such training, collaboration with international partners may help support e-learning initiatives and provide incentives for HCPs to participate [49, 50]. Policy makers should prioritize the development of tailored training programs to enhance system adoption and reduce resistance for change [17, 51]. Forward-looking initiatives, such as MOHP’s National Healthcare Capacity Building Program, aim to enhance HCP skills in Basic Information Technology and Biomedical Informatics, improving data management and decision-making. While the timeline and target number of professionals remain uncertain, this initiative will equip staff with the tools needed to integrate digital health systems like EOHSS [48, 52]. 

Social influence played a minor role in behavioral intention which agrees with several studies on intention to use electronic health records in Ethiopia [39], Cameroon [15], Taiwan [10], USA and Portugal [53]. The weak effect of social influence in the study may be because the EOHSS has not yet been implemented [37]. Support from the surrounding environment, such as peers or official healthcare authorities is important element for dentists’ motivation. In the early implementation stages, social influence may be promoted by opinion leaders or super users within the dental community [54, 55]. These role models advocate for the value of EOHSS, showcasing its benefits and ease of use. Their credibility is a powerful catalyst, encouraging peers to adopt the system. Additionally, networking opportunities allow dentists to share positive experiences and implement training programs to promote acceptance of the system [55]. 

Resistance to change and technology anxiety negatively influenced behavioral intention in agreement with studies conducted in LMICs [18, 27, 28] and theses actors were significant barriers to adopting digital health systems. These challenges are often exacerbated by limited exposure to technology during education and professional practice, leading to a lack of confidence among healthcare workers [56, 57]. Resource constraints, such as inadequate digital infrastructure and technical support, increase apprehension about the reliability and usability of new systems [19, 22, 58]. Additionally, cultural preferences for traditional methods, concerns about increased workload and skepticism about technological benefits can impede acceptance [18, 23]. To mitigate the negative impact, organizations should provide training to empower HCPs, offer accessible technical support and clearly communicate about the value and sustainability of digital health solutions to build trust in new technologies.

There was a significantly stronger positive impact on Behavioral intention among female than male dentists. This aligns with studies on adoption of electronic documents management systems in Kenya [27] and Portugal [59]. This may be attributed to female dentists placing a higher value on ease-of-use because they balance professional and personal responsibilities. Thus, an effortless system may streamline tasks, increasing its acceptance [37, 59]. Also, for dentists over 40 years, effort expectancy had a stronger positive influence on behavioral intention than for dentists < 30 years old. This may because older dentists having established practices, making them less responsive to new systems that require an effort to engage with and more resistant to new disruptive technologies and more skeptic of new systems [15, 23]. Consequently, they place greater importance on effort expectancy to adopt EOHSS [28]. 

### Contextual implications

Egypt has committed to digital transformation in the healthcare sector [44]. Despite the economic challenges facing the country. These challenges may lead to HCPs skepticism regarding new technologies that are costly or not directly related to patient care [43]. EOHSS adoption needs strategies to address barriers related to system purchase and maintenance as well training and incentivizing HCPs. Without this, dentists may be reluctant to invest in training, if the EOHSS sustainability and return on investment are uncertain. It is important to address these financial barriers through collaboration with international partners [60]. Moreover, cultural attitudes toward innovation can influence practitioners’ readiness to embrace new methods, highlighting the importance of peer support. Collaboration among stakeholders, including government and academic institutions, is necessary to promote digital transformation, ultimately improving oral health outcomes in Egypt.

### Strengths and limitations

This study is the first to assess the acceptance of EOHSS in the region. The large sample and diverse geographic coverage add to the study strength. However, the cross-sectional design cannot provide proof of causality and shows only a snapshot of dentists’ readiness. It fails to capture temporal changes or show the dynamic EOHSS adoption process. The use of convenience sampling and online survey may introduce some bias, due to self-selection, potentially over-representing dentists inclined to adopt the EOHSS. These limitations were addressed by recruiting participants with different qualifications and experience levels to increase representativeness. Although the sample is not statistical, it matches the profile of Egyptian dentists in age, gender, and qualification, as reported by the Egyptian Dental Syndicate [61] and Central Agency for Public Mobilization and Statistics [26]. Lastly, the findings are context specific and reflect the situation in Egypt. The contextual factors must be considered when the EOHSS is planned for another country. It is only by reporting the findings in various contexts that the full picture can be seen and that a better understanding can emerge of the possibility to use technology in oral health surveillance in LMICs.

### Future directions

This study focused on key predictors influencing technology adoption within the UTAUT framework, thus enabling the alignment of electronic system features with end users’ needs. Future research should expand to include a wider range of factors, such as organizational preparedness including management, financial, and operational capacity, and regional disparities [62]. Additionally, incorporating constructs such as digital literacy could offer a more thorough understanding of EOHSS adoption. Furthermore, longitudinal studies conducted at different stages of EOHSS deployment, supported by qualitative analyses, would facilitate the monitoring of adoption dynamics and provide deeper insights into the factors influencing EOHSS acceptance.

## Conclusions

The study showed that Egyptian dentists accepted the EOHSS, and this acceptance was associated with performance expectancy, effort expectancy and training adequacy although technology anxiety may hinder its adoption. Effort expectancy had a stronger positive impact among females than males and among older than younger dentists. The acceptance of EOHSS may be improved by strategic planning, managing individual and organizational factors to implement EOHSS in Egypt.

.

## Electronic supplementary material

Below is the link to the electronic supplementary material.

Supplementary Material 1

Supplementary Material 2

## Data Availability

The datasets used and /or analyzed during the current study are available from the corresponding author upon reasonable request.

## Abbreviations

ANX: Anxiety
AVE: Average variance extracted
BI: Behavioral intention
CVI-I: Content validity index for items
EE: Effort expectancy
EOHSS: Electronic oral health surveillance system
FC: Facilitating condition
HCP: Health care practitioner
HIS: Health information system
KMO: Kaiser meyer olkin
m-Oral Health: Mobile oral health
MOHP: Ministry of health and population
NFI: Normal fit index
PE: Performance expectancy
RC: Resistance to change
SEM-PLS: Structural equation modeling using partial least squares model
SEM: Structural equation model
SI: Social influence
SRMR: Standardized root mean square
TA: Training adequacy
UTAUT: Unified theory of acceptance and use of technology
VIF: Variance inflation factor
WHO: World health organization
