# Supplementary material for: Dentists’ readiness to accept an electronic oral health surveillance system in Egypt using a modified framework of the unified theory of acceptance and use of technology (UTAUT): a cross-sectional survey
Source: BMC Oral Health. 2025 Jan 16;25:79. doi: 10.1186/s12903-024-05410-3 (PMC11740471; doi:10.1186/s12903-024-05410-3)
Supplement: Supplementary file 1 — Supplementary Material 1 [file 12903_2024_5410_MOESM1_ESM.docx]

Dentists’ distribution according to geographic location reported in the Annual Bulletin of Health Services Statistics by CAPMAS in 2019 and the proportional distribution of the sample (total n= 1500)

| Geographic Area | Number of dentists | % | Required Sample | Actual responses |
| --- | --- | --- | --- | --- |
| Greater Cairo | 6482 | 31% | 465 | 473 |
| Delta area | 5821 | 28% | 420 | 411 |
| Alexandria area | 2673 | 13% | 195 | 200 |
| Suez Canal | 1948 | 9% | 135 | 119 |
| Upper Egypt | 3910 | 19% | 285 | 267 |
