# Supplementary material for: Dentists’ readiness to accept an electronic oral health surveillance system in Egypt using a modified framework of the unified theory of acceptance and use of technology (UTAUT): a cross-sectional survey
Source: BMC Oral Health. 2025 Jan 16;25:79. doi: 10.1186/s12903-024-05410-3 (PMC11740471; doi:10.1186/s12903-024-05410-3)
Supplement: Supplementary file 2 — Supplementary Material 2 [file 12903_2024_5410_MOESM2_ESM.docx]

**UTAUT Model Questionnaire**

**Section I: Sociodemographic and work-related questions**

1. *Age in years:*
2. 20 – 29
3. 30 – 39
4. 40 – 49
5. 50 – 60
6. *Gender:*
7. Male
8. Female
9. *Educational level:*
10. BDS
11. Diploma
12. MSc
13. PhD
14. *Work Experience in years:*
15. Less than 5
16. 5 - 10
17. >10 - 20
18. More than 20
19. Nature of Primary Practice
20. Primary health care unit
21. General Hospital
22. Teaching Hospital
23. *Healthcare facility geographical location:*
24. Urban
25. Rural

**Section II:** **Modified Unified Theory of Acceptance of Technology (UTAUT) framework for adopting Electronic Oral Health Surveillance System (EOHSS)**

[Scale ranges from strongly disagree to strongly agree]

| **Domains** | **Statements** |
| --- | --- |
| Performance Expectancy (PE) | Using an EOHSS system will: |
|  | PE1: Enable me to record routine patient’s data quickly. |
|  | PE2: Allow me to accomplish more work than would otherwise be possible. |
|  | PE 3: Increase my chances of achieving things that are important to me “or getting a Promotion.” |
|  | PE4: Enable me to make work-related decisions based on better evidence. |
|  | ~~PE5: Increase my productivity~~ |
|  | ~~PE6: Overall, I think EOHSS will be useful in my job.~~ |
| Effort Expectancy (EE) | EE1: Learning to use the EOHSS will be easy for me. |
|  | EE2: My interaction with EOHSS will be understandable and clear. |
|  | EE3: It is easy for me to become skillful at using EOHSS. |
|  | ~~EE4: Overall, EOHSS will be easy to use.~~ |
| Social Influence (SI) | SI1: My coworkers will support learning and the use of EOHSS. |
|  | SI2: My Health facility management team will support learning and the use of EOHSS. |
|  | SI3: The management team of the official authorities responsible for health services will support learning and the use of EOHSS. |
|  | ~~SI4: Overall, people who are important to me will support the learning and use of EOHSS~~ |
| Facilitating Conditions (FC) | FC1: Having resources. (e.g., mobile phone, mobile tablets, Internet) are necessary to use a EOHSS. |
|  | FC2: Having knowledge is necessary to use EOHSS. |
|  | FC3: Prescence of EOHSS experts available at any time for assistance with system difficulties will be very helpful. |
|  | FC4: Having knowledge sources (e.g., manuals, documents) will support my use of EOHSS. |
|  | ~~FC5: Overall, I think that using EOHSS fits well with the way I like to work.~~ |
| Training Adequacy (TA) | TA1: Receiving training on basic use of mobile devices and internet will be very helpful to use the EOHSS efficiently. |
|  | TA2: Training on using EOHSS will be very helpful to use the system efficiently. |
|  | TA3: Presence of a training document as a reference material that I can consult during the use of the EOHSS will be very helpful in using the system efficiently. |
|  | ~~TA4: Overall, receiving training will be very helpful to use EOHSS~~ |
| Behavioral Intention (BI) or Intention to adopt | BI1: I intend to use the EOHSS in the future. |
|  | BI2: I predict I will use the EOHSS in the future. |
|  | BI3: I plan to use the EOHSS in the future. |
| Anxiety towards electronic systems (ANX) | ANX1: I feel nervous about using systems that rely on electronic devices. |
|  | ~~ANX2: The challenge about using electronic devices is exciting~~ |
|  | ANX3: Electronic systems are somewhat intimating to me. |
|  | ~~ANX4: I am able to keep up with technological advances in mobile devices~~ |
|  | ANX5: I feel nervous when using internet-based systems. |
|  | ~~ANX6: It scares me to think I could cause loss of data in the system by hitting the wrong key~~ |
|  | ~~ANX7: I would hesitate to use the system for fear of making mistakes I cannot correct~~ |
| Resistance to change (RC)* | RC1: I don’t want to new electronic system to change the current workflow in the clinic. |
|  | RC2: I don’t want the new electronic system to change the way I record the patients’ data. |

*~~ABC~~: Items struck through were removed from the model due to low item loading values and* Average Variance Extracted *values (AVE) less than 0.50, *Items are reversed coded statements.*
